# Video-assisted thoracic surgery sleeve resection and bronchoplasty using 3D imaging system: its safety and efficacy

**DOI:** 10.1186/s13019-021-01685-7

**Published:** 2021-10-16

**Authors:** Yong Won Seong, Jae Hyun Jeon, Hyo-Jun Jang, Sukki Cho, Sanghoon Jheon, Kwhanmien Kim

**Affiliations:** 1 Department of Thoracic and Cardiovascular Surgery, Seoul Metropolitan Government-Seoul National University Boramae Medical Center, Seoul National University College of Medicine, Seoul, Korea; 2 Department of Thoracic and Cardiovascular Surgery, Seoul National University Bundang Hospital, Seoul National University College of Medicine, Seoul, Korea; 3 Department of Thoracic and Cardiovascular Surgery, Hanyang University Hospital, Seoul, Korea

**Keywords:** Video-assisted thoracic surgery, Lung cancer, Bronchi, Surgical anastomosis, Suture techniques, Imaging, Three-dimensional

## Abstract

**Background:**

Video-assisted thoracic surgery sleeve resection with bronchial anastomosis or bronchoplasty is a technically demanding procedure. Three-dimensional endoscopic surgery has been reported to be helpful in decreasing operation time and improving spatial perception with less surgical errors, but there have been rare reports about relatively difficult thoracoscopic procedures utilizing 3D thoracoscope. We performed this study to evaluate early clinical outcomes of thoracoscopic sleeve resection and bronchoplasty utilizing 3D thoracoscope.

**Methods:**

Data from a total of 36 patients who underwent thoracoscopic sleeve lobectomy or bronchoplasty at our institution from December 2015 to October 2017 were retrospectively reviewed. Three-port approach with one utility incision was used with a 10 mm, 30° three-dimensional thoracoscope. Twenty-three patients (81%) were male, and mean age was 65.9 ± 9.4 years. Fourteen patients (38.9%) underwent sleeve resection with bronchial anastomosis, 22 (61.1%) underwent wedge or simple bronchoplasty, and one patient received concomitant PA procedure. Bronchial anastomosis sites were not covered with viable tissue flaps.

**Results:**

There was no (0%) suture needle injury from spatial misperception during bronchoplasty or sleeve anastomosis. There was no (0%) operative mortality. The pathologic report revealed squamous cell carcinoma (63.9%), adenocarcinoma (19.4%), carcinoid (6.9%), adenosquamous carcinoma (3.4%), and sarcomatoid carcinoma (2.8%). One (2.8%) late mortality was due to systemic recurrence of sarcomatoid carcinoma. There was no (0.0%) anastomotic failure. The mean number of dissected lymph nodes were 27.4 ± 13.2, and mean operation time was 216.8 ± 60.0 min. Median postoperative 24-h drain amount was 315 mL. Median chest tube days and hospital days were 4 and 6, respectively. Two patients (5.6%) had complications greater than Clavien-Dindo grade II—one case of ARDS, and the other case of a delayed bronchopleural fistula.

**Conclusions:**

Thoracoscopic sleeve resection and bronchoplasty utilizing HD 3D thoracoscope is a safe and effective procedure with excellent early clinical outcomes. Further investigation for long-term outcomes will be needed.

## Background

During the past 20 years, Video-assisted thoracic surgery (VATS) has changed the paradigm of thoracic surgery shifting from conventional surgery by thoracotomy to minimally invasive surgery with smaller and lesser incisions [1–6]. Initially, thoracoscopic surgery was more difficult to learn than open surgery and required additional psychomotor skills due to poor-quality two-dimensional images with lack of depth perception and spatial orientation in earlier days [7–9]. As imaging technology developed and evolved, the quality of the two-dimensional image drastically became better, which enabled the surgeons to perform more difficult and complex procedures by laparoscopy and thoracoscopy [1–5, 10, 11]. However, depending only on the two-dimensional image, the difficulty still persists in performing technically demanding procedures such as bronchial sleeve resection and bronchoplasty. Recently, high-definition (HD) 3D thoracoscope have been developed to overcome the shortcomings of the two-dimensional images. This three-dimensional thoracoscope may enable more technically challenging thoracic surgeries, but there have been rare reports. We performed this study to evaluate early clinical outcomes of VATS bronchial sleeve resection and bronchoplasty which were performed using an HD 3D thoracoscopy system.

## Methods

### Patients

From December 2015 to October 2017, a total of 36 patients underwent VATS sleeve resection or bronchoplasty at our institution using three-dimensional (3D) thoracoscope system. Before December 2015 we only had conventional 2D thoracoscope system and the 3D thoracoscope system was unavailable, and we had been performing sleeve resection or bronchoplasty only via conventional thoracotomy. Medical records and data were obtained from our electronic medical record (EMR) system and reviewed retrospectively. Twenty-three patients (80.6%) were male, and the mean age was 65.9 ± 9.4 years. Patients’ characteristics are shown in the Table 1. Three patients (8.3%) underwent neoadjuvant chemotherapy, and one patient underwent definitive chemoradiation therapy before the surgery. The seventh edition of the American Joint Committee on Cancer (AJCC) TNM staging system was used for clinical and pathological staging. One patient underwent surgery for a benign lung lesion.

**Table 1:** Patients’ characteristics

Variables	% (n = 36)
Sex	
Male	80.6 (29)
Female	19.4 (7)
Age (years)	65.9 ± 9.4
cT stage	
cT1a	2.8 (1)
cT1b	11.1 (4)
cT2a	36.1 (13)
cT2b	8.3 (3)
cT3	22.2 (8)
cT4	5.6 (2)
ycT1c	5.6 (2)
ycT3	5.6 (2)
cN stage	
cN0	77.8 (28)
cN1	8.3 (3)
cN2	11.1 (4)
cM stage	
cM0	100 (35)

### Surgical technique

**Fig. 1:**
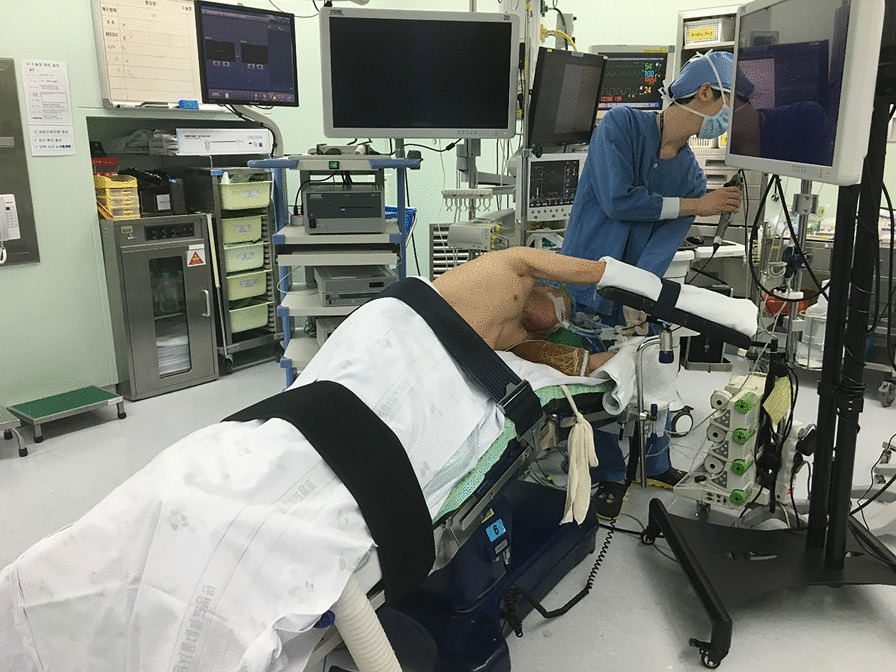
Routine setting of the operating room during our 3D VATS major pulmonary resection. We avoid placement of a central venous catheter and a Foley catheter, but during sleeve resection and bronchoplasty we place a Foley catheter

**Fig. 2:**
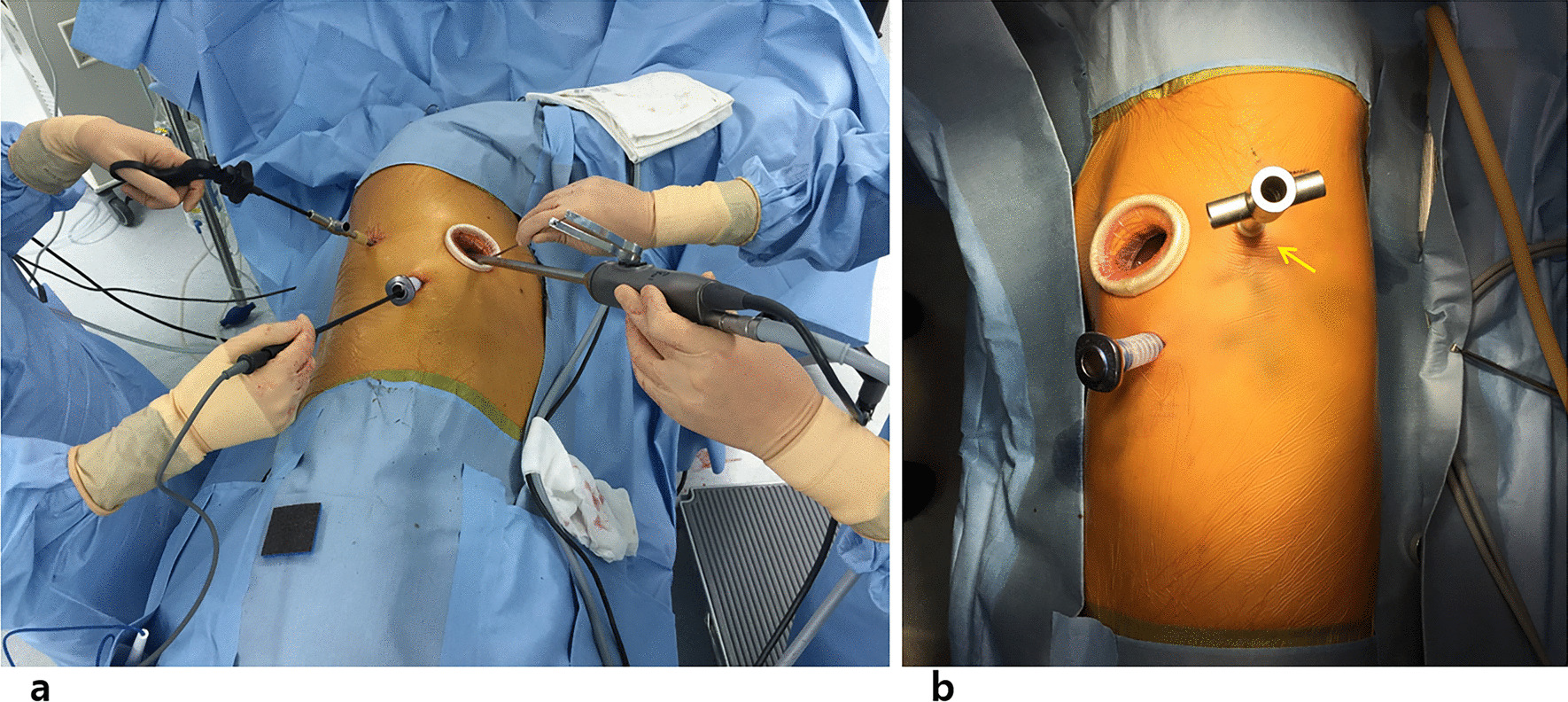
**a** Ports and instruments placement in right-side surgery. The operator always stands at the right side of the patient. Both of the operator’s arms are comfortably and ergonomically placed, **b** Ports in left-side surgery. Note the 10 mm posterior port placed anteriorly to the scapular tip (small arrow), which is different from the right-sided port placement (5 mm, posteriorly to the scapular tip). Sometimes the camera is inserted through this posterior port during subcarinal lymph node dissection, left paratracheal lymph node dissection, or lower lobar sleeve resection and bronchial anastomosis

**Fig. 3:**
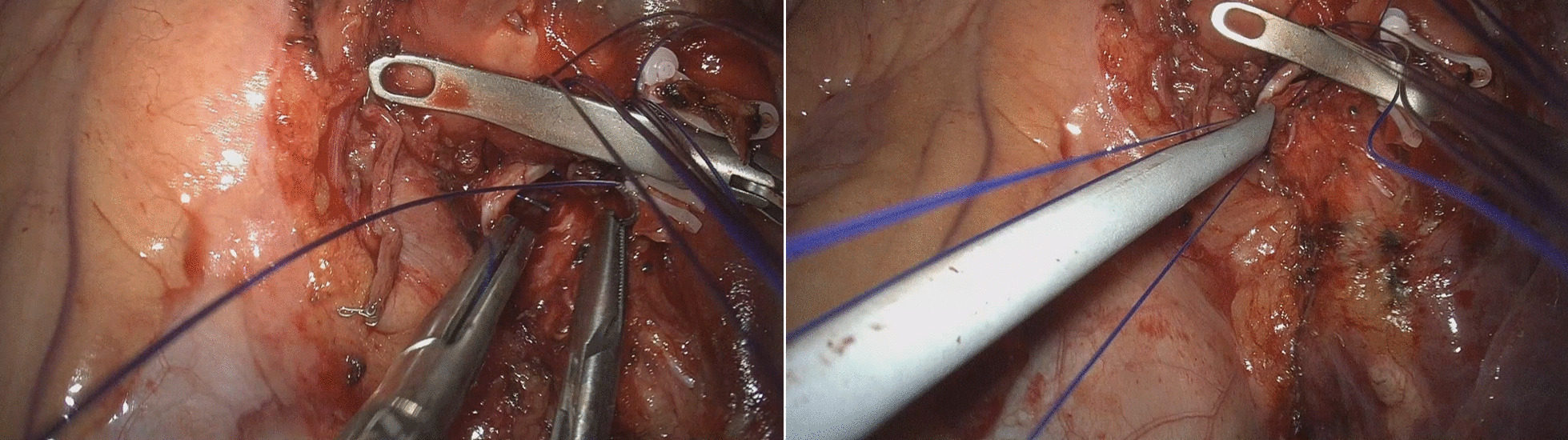
(Left) Bronchial anastomosis after sleeve left upper lobectomy. 3D vision with an endobronchial retracting instrument helps safely performing the anastomosis. (Right) tying down the interrupted sutures with an endoscopic knot-pusher

We have our own early recovery after the surgery (ERAS) protocol for the VATS major pulmonary resections, the essence of which is to avoid placement of a central venous catheter and a Foley catheter. However, we routinely placed a Foley catheter for the sleeve resection and bronchoplasty in this study. The routine scenery of our operating theater is shown in Fig. 1. Two surgeons with enough experience of VATS performed the surgeries. They both used a three-port technique, and one of those ports was a 4 to 5 centimeters long utility incision with an Alexis wound protector (Applied Medical Resources Corp., USA). For right-side surgeries, the posterior port below the scapular tip with a 5 mm trocar and the inferior port at the 7th intercostal space (ICS) on the midaxillary line with a 13mm trocar were used for 5mm instruments and endoscopic staplers, and the thoracoscope camera was mainly inserted through the utility port. For left-side surgeries, the different point is that the posterior port was made just anterior to the scapular tip with a 10 mm trocar, which was used both for the camera and endoscopic instruments, and the other two ports were made and used as same as the right side (Fig. 2). The surgeons always stand at the right side of the patient. This location of the camera at the 5th ICS provides an excellent view of the interlobar area and the hilum and also provides the surgeons an ergonomic position of the instruments to perform the surgery very comfortably. For anesthesia, a left-sided double-lumen endotracheal tubes were used for right-side surgeries, and right-sided double-lumen endotracheal tubes were used for left-side surgeries. A three-dimensional thoracoscope system with a 10mm, 30-degree 3D camera (IMAGE1 S™ 3D camera platform, KARL STORZ SE & Co. KG, Germany) was used, and every participant of the surgery wore a pair of 3D glasses. Pulmonary vessels were divided, mediastinal lymph node dissection was performed, then the endobronchial lesions were carefully and meticulously resected using an endoscopic knife or endoscopic scissors. The specimen was retrieved in an endoscopic bag through the utility incision, then a bronchial anastomosis or a bronchoplasty was performed depending on the extent of the resection. For a bronchoplasty, multiple interrupted 4-0 PDS sutures were made from cephalad to caudal direction, then the sutures were tied one-by-one using an endoscopic knot-pusher (The Endo Slide™ 5 mm knot pusher, Medtronic, Inc.) from caudal to cephalad direction. For a sleeve resection and bronchial anastomosis, deeply placed half of the bronchi was repaired by a continuous running 3-0 Prolene suture, then closer side was repaired by multiple interrupted 4-0 PDS sutures which were similarly made and tied as in a bronchoplasty. Previously made sutures and/or pulmonary artery were meticulously retracted cephalad using a 5mm endoscopic articulating esophageal retractor (Mediflex ^®^, USA). This retractor with the 3D vision enables safe and effective suturing without injury to the pulmonary artery and without mixing and crossing of the previously placed suture materials (Fig. 3). Air-leakage was routinely tested under the two-lung ventilation, then the surgery was finished after insertion of a single 28 Fr. chest tube. Buttressing the anastomosis site with a pedicled flap was not used, but the anastomosis site was covered with a polyglycolic acid sheet (Neoveil, GUNZE LTD., Japan) with the application of fibrin glue (Greenplast, GC Pharma, Korea).

### Statistical analysis

Retrospectively collected data were statistically analyzed using SPSS Statistics, version 20.0 (SPSS, Inc, Chicago, IL). Data were expressed as a mean ± standard error of the mean or median (range) regarding the distribution of the data. Comparisons of the variables were performed using Pearson’s Chi-square test, Fisher’s exact test, and Student’s *t* test.

## Results

Complete resection (R0) was achieved in all the cases. Fourteen patients (38.9%) underwent sleeve resection and anastomosis, three (8.3%) of these patients having lower lobar sleeve resection, the other 11 (30.6%) having upper lobar sleeve resection. Twenty-two patients (61.1%) underwent simple or wedge bronchoplasty. One patient who underwent a left upper lobar sleeve resection received a concomitant pulmonary artery procedure, which was a proximal dissection of the left pulmonary artery and clamping due to a partial invasion of the cancer to the apical truncal branch of the pulmonary artery. Twenty-three patients (63.9%) turned out to have squamous cell carcinoma, which was the most common histology. The mean number of dissected lymph nodes was 27.4 ± 13.2 (0–70), including the zero lymph nodes in the patient who had a benign disease. Postoperative outcomes including pathologic TNM staging is shown in Table 2.

**Table 2:** Postoperative outcomes

Variables	% (n = 36)
Operation	
Sleeve resection and anastomosis	38.9 (14)
Bronchoplasty	61.1 (22)
Concomitant PA procedure	2.8 (1)
Histology	
squamous cell carcinoma	63.9 (23)
Adenocarcinoma	19.4 (7)
Adenosquamous cell carcinoma	2.8 (1)
Carcinoid	5.6 (2)
Sarcomatoid carcinoma	2.8 (1)
Small cell lung cancer	2.8 (1)
No. of dissected lymph nodes (no.)	27.4 ± 13.2 (0–70)
pT stage	
pT1a	13.9 (5)
pT1b	8.3 (3)
pT1c	11.1 (4)
pT2a	27.8 (10)
pT2b	8.3 (3)
pT3	5.6 (2)
pT4	5.6 (2)
ypT1a	2.8 (1)
ypT1c	2.8 (1)
ypT2	5.6 (2)
ypT3	5.6 (2)

There were no (0%) cases of intrathoracic suture needle injury from spatial misperception during bronchoplasty or sleeve anastomosis. There were no cases of operative mortality (0%) nor anastomotic failure (0%). Postoperative bronchoscopy was performed in every patient, which all revealed patent anastomosis without leakage. Mean operation time was 216.8 ± 60.0 minutes. Median postoperative 24-hour drainage amount was 315 mL. Median chest tube days and hospital days were 4 and 6, respectively. Two patients (5.6%) had complications greater than Clavien-Dindo grade II—one case was a patient with ARDS, and the other case was a trivial delayed bronchopleural fistula in the patient who underwent preoperative definitive chemoradiation therapy with a total radiation dose of 66 Gy. The patient who suffered ARDS had to be intubated and went through treatment under mechanical ventilation for five days with IV antibiotics and short-duration steroid. The patient recovered well and could be weaned from the ventilator. This bronchopleural fistula was found three weeks after the surgery and was very small, which spontaneously healed a few days after percutaneous catheter drainage (PCD) insertion without any additional surgery. There was one case (2.8%) of late mortality which was from systemic multiple metastases of sarcomatoid carcinoma. Clinical outcomes are shown in Table 3.

**Table 3:** Clinical outcomes

Variables	% (n = 36)
30-day mortality	0.0% (0)
Late mortality	2.8% (1)
Anastomosis failure	0.0% (0)
Postop 24 h drainage	315 mL (70–780)
Postop 2nd day drainage	260 mL (40–940)
Chest tube duration	4 days (1–30)
Hospital days	6 days (3–31)
Postoperative complications	22.2% (8)
Air-leak > 5 days	8.3% (3)
Pneumonia/ARDS	11.1% (4)
pneumonia	8.3% (3)
ARDS	2.8% (1)
Delayed BPF	2.8% (1)
Subcutaneous emphysema	2.8% (1)

In order to identify the learning curve effect, we compared the mean operation time of the first 12 cases, next 12 cases, and the last 12 cases which were 225.8 ± 47.7, 206.7 ± 72.8, 217.8 ± 60.7, respectively. There was no statistical difference between these groups. In univariate analysis, neoadjuvant radiation was significantly related to postoperative bronchopleural fistula (*P* = 0.034). For the surgeons’ perspective, no surgeons had any single episode of discomfort nor adverse effect after the 3D VATS surgery, such as nausea, dizziness, visual impairment or diplopia. Every surgeon was satisfied with the HD 3D VATS surgery, not wanting to go back to the conventional 2D VATS surgery.

## Discussion

**Fig. 4:**
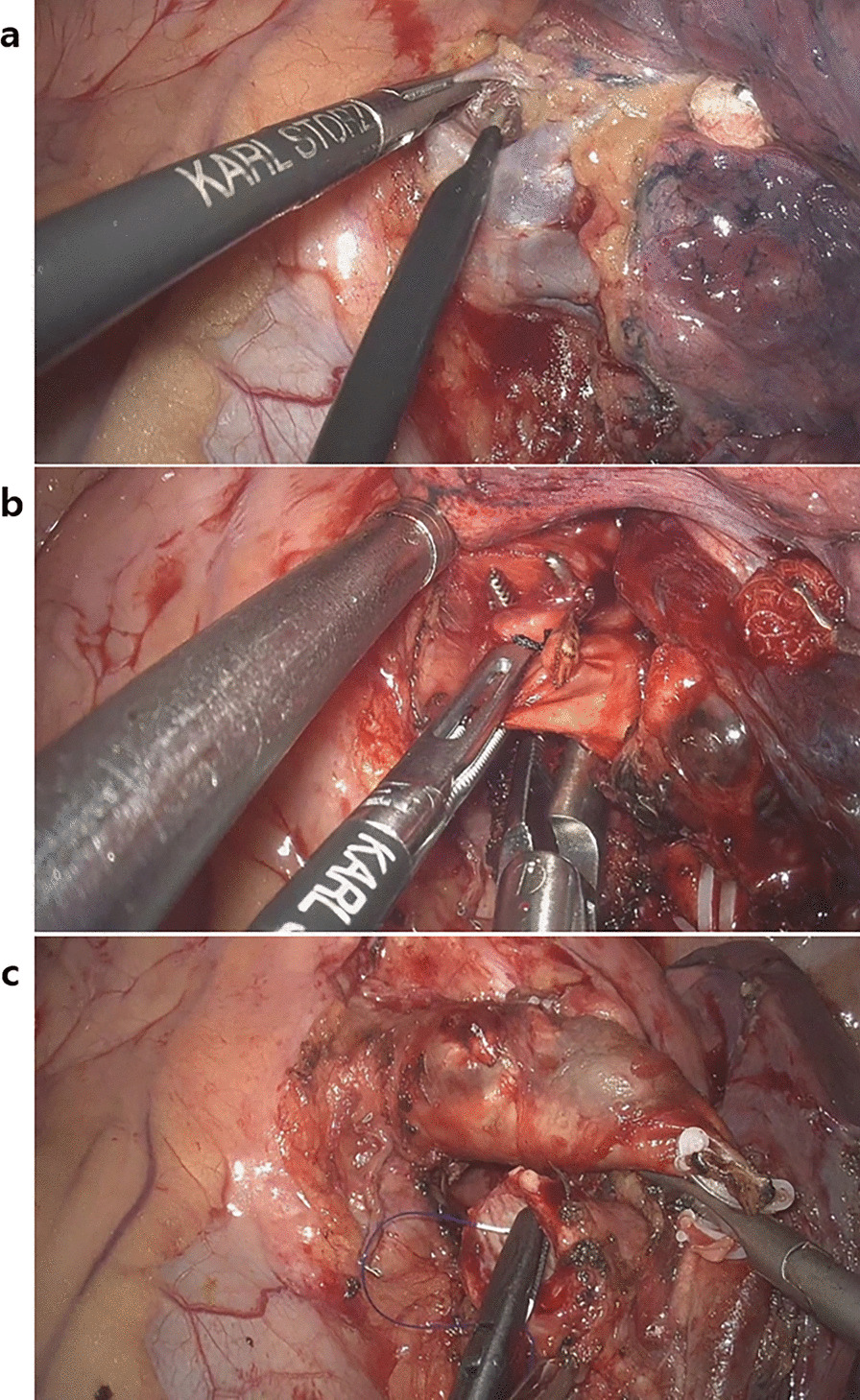
**a** Dissection of the left upper pulmonary vein, showing clear vision of the web-like avascular plane between the pulmonary vein and the underlying left main pulmonary artery, **b** this clear vision of the avascular plane between mediastinal structure enables a safe dissection of the large hilar structures like main pulmonary artery, which is very challenging with a conventional 2D thoracoscope, **c** the HD 3D vision provides the precise direction and orientation of the suture needle, which enables avoidance of any injury to the nearby structures

Pulmonary arteries, veins, bronchi, and mediastinal structures directly abut each other, confined in the hilum and they cross each other structure in three-dimensional fashion with variations. Therefore, pulmonary arteries, veins, and bronchi can be easily injured during the major pulmonary resection. Pulmonary artery and veins are also large arteries which are very close to the heart and if there is bleeding it is devastating. The HD 3D thoracoscope provided us an excellent vision of the avascular plane between the mediastinal structures, therefore, dissecting the vessel felt much easier and safer for us even enabling us to safely dissect and clamp the left main pulmonary artery at the left upper hilum (Fig 4). The HD 3D vision also provided us the precise direction and orientation of a suture needle, enabling us to safely perform the bronchial anastomoses without any vessel injuries (Fig 4).

The development of the thoracoscopic surgery had been similar with the laparoscopic surgery. Adapting to the two-dimensional flat view of the real three-dimensional structures have been one of the greatest challenges in laparoscopic surgery [12]. Therefore surgeons had to relearn these disparities between 3D structures and 2D vision and accustom their brain for spatial orientation [13]. Three-dimensional imaging was first developed as an alternative to the conventional 2D imaging to overcome these shortcomings of the 2D imaging system [14–16]. The 3D laparoscope systems had been introduced more than 20 years ago, but these earlier 3D systems did not have good image quality. The earliest randomized control trial from Hanna et al. using an earlier 3D laparoscope system revealed no advantage over 2D system in laparoscopic cholecystectomy [17]. The following development was the improvement of the quality of the 2D imaging to a higher definition, but Feng et al. reported a better performance with the 3D system than the 2D HD system despite the overwhelming subjective favor towards the 2D HD system [11]. The improvement in the quality of the image for the 3D system followed as well with various higher-definition 3D systems. Wilhelm et al. reported that even well-experienced laparoscopists perform better with 3D systems than with 2D HD systems [10]. Usta et al. reported in their randomized prospective study that a new generation 3D HD laparoscopic system has the potential to shorten the learning curve with reduced operation time and error rate [18]. Recent two systematic reviews reported that 3D laparoscopy was superior or equal to 2D laparoscopy [19, 20]. Komaei et al. also reported from their systematic review that 3D imaging system tends to shorten the operative time compared to 2D systems and result in a better depth perception [21]. The most recent meta-analysis by Liang et al. included 23 articles of which 7 were thoracoscopic and 16 were laparoscopic surgeries. Their analysis revealed that 3D system is superior to the 2D system in clinical settings with significantly shorter operating time, less blood loss and shorter hospital stay [22].

In the aspect of the long-term oncologic outcome, 2D VATS lobectomy is currently regarded to have a better or comparable long-term oncologic outcome with better short-term postoperative outcome in early-stage lung cancer [4, 5, 23–25]. However, there still is controversy in performing VATS lobectomy in patients with more advanced lung cancer due to the shortcomings of the 2D VATS [4].

Despite this improvement in 3D vision technology and increasing positive outcomes from various reports, 3D thoracoscopy is still not the common standard technique for the VATS. There have been a few reports about 3D thoracoscopic surgery that revealed good clinical outcomes after pulmonary resection using a 3D VATS system, and all these reports showed shorter operation time [26–28]. Most of these previous reports regarding 3D laparoscopic surgery and 3D thoracoscopic surgery were about surgical resection or simple surgical procedure, and there have not been many reports about complex surgical resection and reconstruction which require more surgical precision than surgical resection. Bronchial sleeve resection is a complex surgical resection and reconstruction. Our study showed excellent clinical outcomes after bronchial sleeve resection and bronchoplasty using the HD 3D VATS system, which is technically challenging for many of the thoracic surgeons. Huang et al. reported excellent clinical outcomes after three-port VATS bronchial sleeve lobectomy in 118 patients using 2D HD VATS system, and Gonzalez-Rivas et al. also reported a series of uniportal VATS bronchovascular sleeve resections using 2D HD VATS system [2, 29, 30]. Regarding clinical outcomes from 2D VATS sleeve lobectomy versus sleeve lobectomy via thoracotomy, 2D VATS lobectomy revealed favorable clinical outcomes from many reports. Zhong et al. reported from their systematic review and meta-analysis that 2D thoracoscopic sleeve lobectomy is a safe and efficient procedure with comparable clinical outcomes and oncologic results compared to thoracotomy sleeve lobectomy [31]. Deng et al. reported from their recent meta-analysis that 2D VATS sleeve lobectomy yielded less surgical trauma than thoracotomy sleeve lobectomy and improved postoperative recovery without compromising oncological prognosis. The clinical outcomes of 2D VATS sleeve lobectomy from the five included studies of the meta-analysis revealed number of lymph nodes varying from 10 ± 3.7 to 25.7 ± 6.5, operation time varying from 198.8 ± 58.3 to 300.3 ± 71.7 minutes, postoperative hospital stay varying from 5.7 ± 2.2 to 11.6 ± 2.8 days, and complication rate varying from 11.1 to 35.7% [32]. Since we only performed sleeve resection or bronchoplasty only by VATS, we were not able to compare the clinical outcomes from 3D VATS group and those from 2D VATS group. Our data revealed number of lymph nodes of 27.4 ± 13.2, operation time of 216.8 ± 60.0 minutes, hospital stay of median 6 days, and complication rate of 5.6%, which can be considered comparable from the previously reported excellent clinical outcomes from the 2D VATS sleeve lobectomy [31, 32]. Uniportal VATS is gaining popularity these days but is reported to have some early difficulty and require a certain amount of learning curve because there is only one utility incision to share for the operator and the assistants [33–36]. However, our data of the operating time by cases revealed that 3D HD VATS system might have provided us a quick adaptation which does not require much learning curve. We used the three-port technique with the camera viewing from the 5th ICS utility incision which provides a very nice view of the hilum and the surgeons’ arms are positioned in a very comfortable and ergonomic way, causing less fatigue. In our series, all the surgeons were satisfied without any subjective complaints or adverse effects. This 3D HD VATS system also may have a positive influence such as decreasing the learning curve on the area of the uniportal thoracoscopic surgeries in the future.

We did not routinely cover the bronchial anastomosis or bronchoplasty sites with viable flaps, and one patient from our series suffered from a small delayed bronchopleural fistula at the anastomosis site. Covering the anastomosis site with a viable tissue flap is reported to prevent the bronchopleural fistula [37, 38]. Our data suggest that it would be beneficial to cover the anastomosis site with a viable flap if the patient had undergone chemoradiation therapy before the operation.

The single-cohort retrospective design with a small number of patients is the limitation of our study. A long-term oncologic outcome is must be analyzed later. Further prospective randomized clinical trials comparing 2D HD VATS and 3D HD VATS system might be needed to prove the benefits of the 3D HD system.

## Conclusions

Thoracoscopic sleeve resection and bronchoplasty utilizing HD 3D thoracoscopy system is a safe and effective procedure with excellent early clinical outcomes without any anastomotic failure or 30-day mortality. Further investigation for comparing 2D VATS and 3D VATS and for long-term oncologic outcome is needed.

## Data Availability

The datasets generated and/or analysed during the current study are not publicly available due to our patients’ privacy policy but are available from the corresponding author on reasonable request.

## Abbreviations

VATS: Video-assisted thoracic surgery
HD: High-definition
AJCC: American Joint Committee on Cancer
EMR: Electronic medical record
ICS: Intercostal space
ERAS: Early recovery after the surgery
